# Novel pathogenic *FOXP3* variant in two unrelated families with IPEX syndrome

**DOI:** 10.70962/jhi.20250036

**Published:** 2026-11-10

**Authors:** Ina C.D. Schim van der Loeff, Matthew B. Johnson, David I. Campbell, Eleri Williams, Sophie Hambleton

**Affiliations:** 1 Newcastle University Translational and Clinical Research Institute, Newcastle upon Tyne, UK; 2 Paediatric Immunology and Infectious Diseases, Great North Children’s Hospital, Newcastle upon Tyne Hospitals NHS Foundation Trust, Newcastle upon Tyne, UK; 3 Clinical and Biomedical Science, Faculty of Health and Life Sciences, University of Exeter, Exeter, UK; 4 Paediatric Gastroenterology, Great North Children’s Hospital, Newcastle upon Tyne Hospitals NHS Foundation Trust, Newcastle upon Tyne, UK

## Abstract

The authors describe three patients with IPEX due to a novel pathogenic variant, two of which have been successfully treated with stem cell transplant. Confidence about variant pathogenicity is difficult in IPEX, and hematopoietic stem cell transplantation (HSCT) outcomes are variable; reporting classical IPEX due to novel variants, in this case with an elevated TSDR, remains essential.

Forkhead box P3 (FOXP3) is the master transcription factor of regulatory T cells (Treg, CD4^+^CD25^hi^CD127^lo^), and pathogenic variants in *FOXP3* are known to cause IPEX (immune dysregulation, polyendocrinopathy, enteropathy, X-linked) syndrome (1). Typical manifestations of IPEX include early-onset autoimmune enteropathy, type 1 diabetes mellitus, and dermatitis, typically atopic eczematous dermatitis (1). The disease can present neonatally, and both miscarriages and hydrops fetalis are also associated with impaired function of FOXP3, consistent with in utero autoimmunity (1). Clinical phenotypes are highly variable, even among patients with identical genetic lesions, which can delay diagnosis and complicate therapeutic decision-making. Over 70 variants in *FOXP3* have been described as causing IPEX, many located in the forkhead DNA-binding domain (1). Complete absence of FOXP3 expression results in the most severe IPEX phenotypes, although most disease-causing variants in *FOXP3* do not affect its expression but rather its function (1). Many patients with IPEX, in fact, have identifiable FOXP3-expressing CD4^+^CD25^hi^CD127^lo^ cells by flow cytometry, although these Treg-like cells lack suppressive function (1). The presence of these Treg-like cells, as well as preservation of FOXP3 expression, complicates the diagnosis of IPEX. The assessment of demethylation at the Treg-specific demethylated region (TSDR) within the CD4 T cell effector compartment (TSDR/CD4 ratio) can be a useful adjunct to distinguish IPEX from other Tregopathies (1). IPEX treatment consists of supportive management including parenteral nutrition, replacement therapy in case of endocrine autoimmunity, and immunosuppression, typically with a regimen containing sirolimus (also known as rapamycin). The only curative therapy for IPEX is currently allogeneic hematopoietic stem cell transplantation (HSCT), and early transplant is associated with superior outcomes (2).

We recently identified the same missense variant in *FOXP3* in three male infants—two siblings and an unrelated male infant (Fig. 1 A). The first (A1) presented with insulin-dependent diabetes at 13 wk of age before developing hypothyroidism and diarrhea, presumed due to autoimmune enteropathy. He died of pneumonia at 10 mo of age. Retrospective targeted next-generation sequencing of disease genes linked to congenital or early-onset autoimmune diabetes (https://www.diabetesgenes.org) identified the c.991T>G, p.(Phe331Val) *FOXP3* missense variant, which was absent from gnomAD 4.0 (SCV007681017). The resulting amino acid change is predicted deleterious in silico and involves a conserved phenylalanine residue in the leucine-zipper forkhead loop close to a cluster of known pathogenic variants (1).

**Figure 1.**
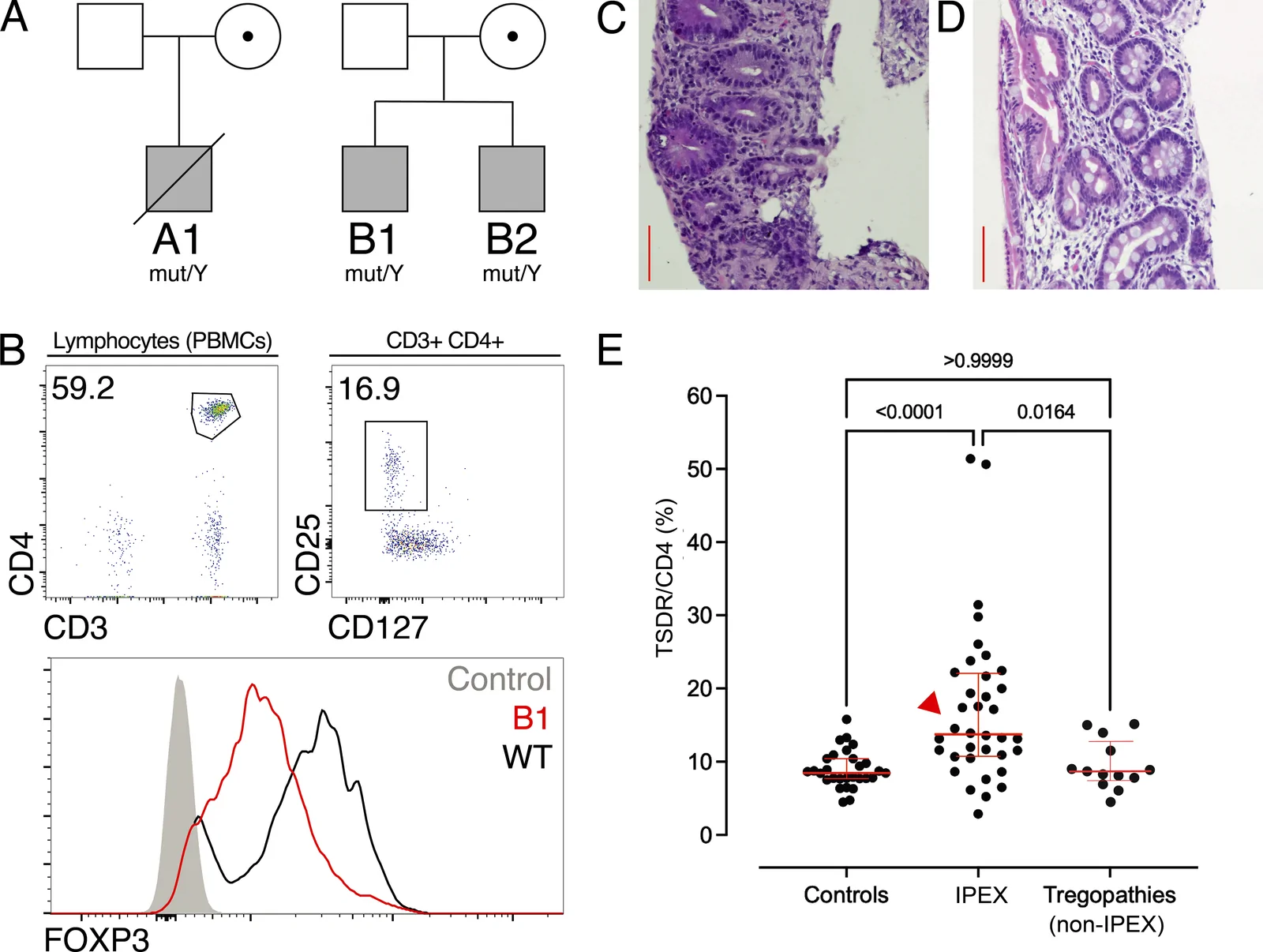
***FOXP3* c.991T>G is a pathogenic variant with respect to IPEX syndrome. (A)** Family tree of the patients presenting with infant-onset diabetes (A1) and autoimmune enteropathy (B1, B2). **(B)** CD25^+^CD127^lo^ Treg and FOXP3 expression by flow cytometry (B1) (using Human Regulatory T Cell Staining Kit, eBioscience 88–8999 and BD CD4 PE-Cy7 348809, BD CD3 FITC 345764, BD CD25 PE 341011, BD CD127 Alexa Fluor 647 558598, and CD25 APC 340907). PBMCs, peripheral blood mononuclear cells. **(C and D)** Gut histology (B1) pre- and posttransplant (automated hematoxylin and eosin staining with Dako Coverstainer and images acquired with Nikon Eclipse Ni microscope) (scale bar in red 100 µm). **(E)** TSDR/CD4 demethylation ratio, measured and calculated as previously described (3) (A1 marked with red arrowhead).

Independently, the same *FOXP3* variant was identified in brothers with a compatible clinical history for IPEX, lending further weight to its pathogenicity. Infant B1, the first child of unrelated parents, presented at 5 wk of age with a 2-wk history of diarrhea, metabolic acidosis, and weight loss (being 20.4% below birth weight). He was resuscitated, treated with empiric antibiotics, and started on parenteral nutrition and gut rest. Endoscopic biopsies demonstrated villous atrophy, apoptosis, and mucosal inflammation suggestive of autoimmune enteropathy (Fig. 1 C). Accordingly, symptoms improved with immunosuppression using high-dose steroids followed by sirolimus. He was euglycemic throughout despite a raised anti-GAD (glutamic acid decarboxylase) antibody titer, which dropped as immunosuppression was initiated. Of note, his mother did not have detectable anti-GAD antibodies. There were periods of dry skin or mild eczema, but these were not a prominent feature of his presentation. Flow cytometric analysis of lymphocyte subsets appeared normal, including detectable CD4^+^CD25^hi^CD127^lo^ cells. Treg FOXP3 expression was detectable by flow cytometry, albeit with reduced intensity (Fig. 1 C). The rare maternally inherited missense variant in *FOXP3* was identified on whole-genome sequencing, initially reported as a variant of unknown significance but later reclassified as Likely Pathogenic in line with Association for Clinical Genomic Service guidelines, taking into account in silico predictions of pathogenicity, rarity of the missense variant, severity of the patient phenotype, and evidence of increased TSDR/CD4 demethylation ratio in A1 (Fig. 1 E). B1 received a matched unrelated donor (MUD) peripheral blood HSCT at 8 mo of age following standard (alemtuzumab, fludarabine, treosulfan, and thiotepa) conditioning and with cyclosporine and mycophenolate mofetil as graft-versus-host disease prophylaxis. He had few complications, was tolerating full enteral feed from 1 mo after HSCT, and his duodenal histology had normalized completely at 7 mo after HSCT (Fig. 1 D). At the last follow-up, 28 mo after HSCT, B1 was thriving (50th centile) with 100% whole blood chimerism, normal naive T cell numbers (CD4 480, CD8 412 cells/mm^3^), and normal immunoglobulins off replacement (IgA 0.5, IgG 6.4, IgM 0.56 g/L). He has tolerated and responded to his first set of post-HSCT vaccinations.

The third infant (B2), brother to B1, was diagnosed in utero as carrying the same variant on amniocentesis, which also enabled human leucocyte antigen typing and putative donor identification. He was born in good condition at term with some mild dry skin but developed loose stools at 3 wk with a mildly raised fecal calprotectin (814 µg/g). Endoscopy showed evidence of early autoimmune enteropathy with intraepithelial lymphocytes, and he was started on sirolimus. At 8 wk of age, he received a fully conditioned MUD HSCT without complications. At last follow-up, 20 mo after HSCT, B2 was thriving, engrafted with 100% whole blood chimerism and normal naive T cell numbers (CD4 932, CD8 932 cells/mm^3^). Like his brother, he has stopped immunoglobulin replacement and has started his post-HSCT vaccination. Of note, B1 and B2’s mother and grandmother are both carriers of the c.991T>G, p.(Phe331Val) *FOXP3* missense variant.

Interestingly, the TSDR/CD4 demethylation ratio for A1 had previously been reported as part of a larger cohort of patients with early onset diabetes and at 15.97% was >95th centile of controls (3) (Fig. 1 E). The TSDR is a conserved CpG-rich region within the *FOXP3* locus. It is progressively demethylated during Treg commitment in the thymus (4). Patients with IPEX have normal or elevated TSDR, consistent with normal lineage differentiation of Treg cells that are “unstable” and give rise to CD4 effector T cells (5). The TSDR demethylation/CD4 ratio does not correlate with the proportion of Treg in patients with IPEX, but an elevated TSDR/CD4 demethylation ratio seems characteristic of IPEX in two cohorts reported so far (3). In both cohorts, there is a suggestion that TSDR demethylation might correlate with disease activity and severity.

Taken together, the clinical presentation, genetic and epigenetic evidence in the three infants described strongly suggest the c.991T>G (p.(Phe331Val)) variant is pathogenic for IPEX syndrome. Assigning variant pathogenicity in patients with IPEX remains difficult given clinical heterogeneity, particularly when variants are hypomorphic. While the TSDR/CD4 demethylation ratio should not guide clinical management in isolation, our experience attests that epigenetic tests may be a useful adjunct to clinical, immunological and genetic results. This test is considerably easier to perform than in vitro Treg suppression assays, which require fresh blood, are affected by immunosuppressive treatments and are typically only carried out in specialized research laboratories. Furthermore, the cases above highlight the importance of early recognition, diagnosis, and therapy of IPEX syndrome to achieve good clinical outcomes. Overall survival after HSCT for IPEX is 73%, while disease-free survival is only 60%, and patients with significant organ damage had the poorest survival even when receiving reduced-intensity conditioning (2). It is clear that early HSCT can be associated with good outcomes, as was the case for two of the patients reported here.

## Ethics statement

### Ethics approval

Written informed consent was provided by all human subjects or their legal guardians in accordance with the 1975 Helsinki principles for enrollment in research protocols that were approved by the Newcastle and North Tyneside Research Ethics Committee 1, UK (REC reference 16/NE/0002 and 20/NE/0044) or the North Wales Research Ethics Committee, UK (IRAS project ID 231760).

## Data Availability

Sequencing data that support the findings of this study are available on request from the authors.
